# CCT6A facilitates lung adenocarcinoma progression and glycolysis via STAT1/HK2 axis

**DOI:** 10.1186/s12967-024-05284-7

**Published:** 2024-05-15

**Authors:** Shao-Kun Yu, Tao Yu, Yu-Ming Wang, Ao Sun, Jia Liu, Kai-Hua Lu

**Affiliations:** 1 Department of Oncology, The First Affiliated Hospital of Nanjing Medical University, Nanjing, China; 2 Department of Pathology, Peking Union Medical College Hospital, Chinese Academy of Medical Sciences and Peking Union Medical College, Beijing, China

**Keywords:** CCT6A, Lung adenocarcinoma, Aerobic glycolysis, HK2, STAT1

## Abstract

**Background:**

Chaperonin Containing TCP1 Subunit 6 A (CCT6A) is a prominent protein involved in the folding and stabilization of newly synthesized proteins. However, its roles and underlying mechanisms in lung adenocarcinoma (LUAD), one of the most aggressive cancers, remain elusive.

**Methods:**

Our study utilized in vitro cell phenotype experiments to assess CCT6A’s impact on the proliferation and invasion capabilities of LUAD cell lines. To delve into CCT6A’s intrinsic mechanisms affecting glycolysis and proliferation in lung adenocarcinoma, we employed transcriptomic sequencing and liquid chromatography-mass spectrometry analysis. Co-immunoprecipitation (Co-IP) and chromatin immunoprecipitation (CHIP) assays were also conducted to substantiate the mechanism.

**Results:**

CCT6A was found to be significantly overexpressed in LUAD and associated with a poorer prognosis. The silencing of CCT6A inhibited the proliferation and migration of LUAD cells and elevated apoptosis rates. Mechanistically, CCT6A interacted with STAT1 protein, forming a complex that enhances the stability of STAT1 by protecting it from ubiquitin-mediated degradation. This, in turn, facilitated the transcription of hexokinase 2 (HK2), a critical enzyme in aerobic glycolysis, thereby stimulating LUAD’s aerobic glycolysis and progression.

**Conclusion:**

Our findings reveal that the CCT6A/STAT1/HK2 axis orchestrated a reprogramming of glucose metabolism and thus promoted LUAD progression. These insights position CCT6A as a promising candidate for therapeutic intervention in LUAD treatment.

**Supplementary Information:**

The online version contains supplementary material available at 10.1186/s12967-024-05284-7.

## Introduction

Lung cancer stands as the second most common cancer worldwide and the foremost cause of cancer mortality [1]. Approximately 85% of diagnosed lung cancers are non-small cell lung cancers, and lung adenocarcinoma (LUAD) is the most common subtype of them [2]. Due to its high malignancy and the limited effectiveness of chemotherapy, patients with LUAD faced grim survival outcomes. The advent of targeted therapies has markedly prolonged the survival of patients with mutations in key driver genes such as EGFR, ALK, MET, ROS1, RET, and HER2 [3]. Nevertheless, a significant fraction of LUAD patients do not possess these mutations, highlighting the critical need for identifying new actionable driver genes [4–6].

Molecular chaperones, responsible for the correct folding of oncogenic proteins, are vital for the advancement of LUAD [7–9]. In eukaryotic cells, the normal folding process is facilitated by chaperone proteins, notably heat shock protein 60 (HSP60) and chaperonin-containing TCP1 (CCT) complex [10]. CCT, consisting of eight homologous subunits (including TCP1, CCT2, CCT3, CCT4, CCT5, CCT6A/B, CCT7, and CCT8) [11], is essential in folding about 10% of cytoplasmic proteins including actin, tubulin [12], CDC20 [13], YAP [14] and STAT3 [15]. CCT subunits prevent misfolding and aggregation, enabling nascent or inactivated proteins to efficiently adopt their intrinsic functional conformation [16]. Abnormal CCT expression protein can lead to disrupted tumor cell metabolism and apoptotic signaling, fostering tumorigenesis. For instance, CCT2 has been found to regulate metastasis and drug resistance in malignant tumors like breast cancer and glioma [16, 17]; the expression of CCT4 and CCT6A can be utilized in the prognostic analysis of liver cancer [18]. Yet, the impact of CCT and its components on LUAD remains unclear.

Abnormal metabolism is a hallmark feature of cells during tumor initiation and progression [19]. The phenomenon of active glycolysis in the presence of ample oxygen is referred to as “aerobic glycolysis” [20]. Aerobic glycolysis enables swift ATP generation to cater to the heightened demands of cancer cells during rapid growth and proliferation. Additionally, it supplies essential intermediate metabolites crucial for nucleotide, amino acid, and lipid synthesis [21]. Numerous studies have substantiated the correlation between cancer and various glycolytic enzymes [22–25]. Hexokinase2 (HK2) catalyzes the conversion of glucose to glucose-6-phosphate, a key rate-limiting step in glycolysis and energy production [26, 27]. Its expression is significantly upregulated in cancer cells compared to normal cells, and it is closely associated with rapid proliferation and drug resistance in diverse cancer types [28–31].

Our study revealed that CCT6A facilitated the stabilization of the STAT1 protein, which in turn transcriptionally upregulates HK expression, thereby enhancing aerobic glycolysis and promoting LUAD cell progression. This suggests that the metabolic reprogramming induced by CCT6A could advance LUAD progression, making the CCT6A/STAT1/HK2 axis a promising target for therapeutic intervention.

## Materials and methods

### Bioinformatic analysis and clinical specimens

Public mRNA sequencing data and corresponding survival data of LUAD were downloaded from TCGA (https://cancergenome.nih.gov/) and GEO (https://www.ncbi.nlm.nih.gov/). We obtained the LUAD tissue microarray (TMA) (AF-LucSur2202) from Hunan AiFang Biological and the final follow-up time was September 2022. A total of 80 LUAD tissue specimens and 80 paired normal tissue specimens were collected to compose the TMA. Immunohistochemistry (IHC) was performed with an anti-CCT6A antibody (AiFang, China, AF01764) and H-score was calculated by the formula: H-score = expressing intensity × expressing percentage. This study was approved by the Institutional Review Board of the First Affiliated Hospital of Nanjing Medical University (2023-SRFA-076).

### Cell lines

The human LUAD cell lines A549 (FH0045), PC9 (FH0083), H23 (FH0616), H1975 (FH0086), and H1299 (FH0908), as well as the human normal pulmonary epithelial cell line BEAS-2B (FH0319), were obtained from FuHeng Biology Company (Shanghai). All cells were cultured in RPMI 1640 medium (GIBCO, USA), supplemented with 10% fetal bovine serum (FBS, GIBCO, USA) and 100U/mL penicillin and 100ug/ml streptomycin (GIBCO, USA), at 37 °C in a 5% CO2 atmosphere.

### Transfection

Specifically synthesized siRNA and shRNA were provided from Shanghai Genechem company (sequence shown in Table S1). Full length CCT6A and HK2 cDNA was cloned into the pLVX-FLAG-puro vector for CCT6A and HK2 overexpression. Transfection was performed utilizing Lipo8000™ Transfection Reagent (Beyotime, China) according to the manufacturer’s protocol. The stably transfected cells were selected out with puromycin at a density of 5 µg/ml.

### Western blotting (WB) and antibodies

Total protein was obtained using RIPA lysis buffer (Beyotime, P0013B, China) containing protease inhibitor cocktails (Beyotime, ST506, China), and the concentration was measured using the BCA protein assay kit (Beyotime, P0012, China). Subsequently, 10 µg of each protein sample was separated by 10% SDS-polyacrylamide gelelectrophoresis (SDS-PAGE), transferred onto polyvinylidene fluoride (PVDF) membranes and then blocked with 5% skim milk powder in Tris-buffered saline with Tween 20 (TBST) for 2 h at room temperature. The membranes were then incubated with primary antibodies overnight at 4 °C, followed by horseradish peroxidase (HRP)-conjugated secondary antibodies for 2 h at room temperature. The protein levels were detected with an enhanced chemiluminescence reagent (xinsaimei, China). Antibodies involved were: CCT6A (Proteintech, China, 19793-1-AP), STAT1 (Santa Cruz, USA, sc-464), β-Actin (CST, USA, #3700), HK2 (Proteintech, China, 66974-1-Ig), and their dilution rates were all 1:1000.

### RNA extraction and quantitative real-time PCR (qRT-PCR)

Total RNA lysis was obtained using an RNA extraction kit (Beyotime, R0027, China). Reverse transcription was performed using HiScript III RT SuperMix for qPCR (+ gDNA wiper) (Vazyme, R323-01, China) with 1 µg of total RNA. Amplification of synthesized cDNA was performed using ChamQ SYBR qPCR Master Mix (High ROX Premixed) (Vazyme, Q341-02, China). β-actin was used as an internal control to normalize gene expressions. The relative RNA levels of the indicated genes were calculated using the 2^−ΔΔCT^ method. Each experiment was performed in triplicate. The primers used were also shown in Table S1.

### Cell proliferation and apoptosis assay

For the colony formation assay, cells were seeded in 6-well plates at a density of 1500 cells per well. After 7–10 days, cells were washed with PBS twice, fixed with 4% paraformaldehyde for 20 min, and then stained with crystal violet (Beyotime, C0121, China) for 20 min. Colonies were photographed and counted with ImageJ.

For the CCK-8 assay, cells were seeded in 96-well plates at a density of 2500 cells per well. Optical density (OD) values were measured at 450 nm with a CCK-8 assay (Beyotime, C0038, China) every 24 h for 96 h.

For cell apoptosis measurement, 1 × 10^5^ cells were collected and washed with PBS twice. Then, the cells were labeled with the Annexin V-FITC/PI apoptosis detection kit (Beyotime, C1062M, China) following the instructions. The samples were then analyzed with fluorescence activated cell sorter (FACS). Annexin V(+)/PI(−) represented early apoptosis while Annexin V(+)/PI(+) denoted late apoptosis.

### Cell migration assays

3 × 10^4^ cells in 300 µl of serum-free medium were added to the upper chamber while medium with 10% FBS was added to the lower chamber. After 24 h incubation, migrating cells were fixed with 4% paraformaldehyde for 20 min and stained with crystal violet for 20 min. Then, the cells were counted at 10×magnification. Subsequentially, cells were seeded in twelve-well plates for the wound healing assay. Scratches were made using a 1000 µl pipette tip after adherence and 95% coverage. After 48 h incubation, bright-field images of randomly selected views were captured at 10×magnification. The cell migration rate was calculated using the formula: [1-(remained wound area/initial wounded area)×100%].

### In vivo xenograft model

Animal studies were approved by the Laboratory Animal Welfare and Ethics Committee of the First Affiliated Hospital of Nanjing Medical University. About 1 × 10^6^ cells were suspended in 100 µl PBS and then injected subcutaneously into BALB/c nude mice (5 weeks old, Gempharmatech, D000521). Every 4–5 days, tumor volume was measured and calculated using the following equation: (longer axes×shorter axes^^2^)/2. Tumor weight was calculated after executed and then fixed in 4% formalin for IHC analysis. All animal experiments were approved by the Committee on the Ethics of Animal Experiments of the Nanjing Medical University (IACUC-2,310,034).

### Glucose consumption, lactic acid production, and intracellular ATP detection

Glucose consumption and lactic acid production were measured using fluorescence-based assay kits as described by the manufacturer’s instructions (Nanjing Jiancheng Bioengineering Institute, F006-1-1, A019-2-1, China) respectively. Intracellular ATP levels were determined using an ATP luminometric assay kit (Beyotime, S0027, China) according to the manufacturer protocol. The total amounts of glucose consumption, lactate acid production, and intracellular ATP were normalized to protein concentration.

### Proton efflux rate (PER)

PER was determined using a Seahorse XFe96 analyzer (Seahorse Bioscience, Agilent Technologies). 1*10^4^ stable transfected cells per well were seeded into 96-well XF cell culture microplates. After 24 h incubation, the medium was replaced by Seahorse XF RPMI medium (pH 7.4) containing glucose (10 mM), glutamine (2 mM), and sodiumpyruvate (1 mM). Subsequently, cells were respectively treated with rotenone/antimycin A (0.5 µM) and 2-deoxy-d-glucose (50mM).

### RNA-sequencing (RNA-seq)

RNA sequencing was conducted using the Illumina HiSeq^TM^2500 system by BGI Genomics (Beijing, China). Initially, mRNA was isolated and enriched with oligo(dT)-attached magnetic beads. The mRNA was then fragmented, and cDNA synthesis followed. Subsequently, PCR was performed and the PCR products were circularized to form single-stranded circular DNA molecules. These circular DNA molecules were replicated via rolling cycle amplification to generate DNA nanoballs (DNBs), which were loaded into nanoarrays and sequenced using the combinatorial Probe-Anchor Synthesis (cPAS) method.

### Co-immunoprecipitation assays (Co-IP)

Total protein was obtained using RIPA lysis buffer (Beyotime, P0013B, China) containing protease inhibitor cocktails (Beyotime, ST506, China). Protein lysates were then incubated overnight with primary antibodies or IgG and Protein A/G PLUS-Agarose (Santa Curz, sc-2003, USA). The beads were then washed with PBS, and resuspend in SDS-PAGE buffer. After centrifuged, the supernatant of the resuspended protein was detected using WB. For ubiquitination assays, A549 cells were treated with MG132 (10µM, 8 h), and the cell lysates were immunoprecipitated by STAT1 antibody, and STAT1 ubiquitination was analyzed by WB with Poly-ub antibody.

### Mass spectrometry analysis

Co-immunoprecipitated proteins were visualized using a Coomassie Blue Staining Kit (Beyotime, China). The protein bands of interest were meticulously excised from the gel and subsequently digested. These peptides were analyzed with nanoLC-MS/MS (Sangon Biotech, Shanghai, China), using an Orbitrap Fusion mass spectrometer. The mass spectrometry-generated raw spectral data were then processed and analyzed employing PEAKS Studio 8.5 software for detailed characterization.

### Immunofluorescence assay

The immunofluorescence assay followed the directions of Immunol Fluorescence Staining Kit (Beyotime, China). Cells were fixed by 4% paraformaldehyde for 20 min, permeabilized for 10 min by 0.5% Triton X-100 and blocked for 1 h with blocking buffer. Subsequently, cells were incubated with primary antibodies overnight at 4 °C and followed by incubation with fluorescent secondary antibodies for 2 h in the dark at room temperature. DAPI was used for nuclear staining. Fixed cells were then photographed to show the distribution of proteins at a 40× magnification.

### Chromatin immunoprecipitation (ChIP) assay

A ChIP assay was performed using a ChIP Assay Kit with Protein A/G Magnetic Beads (Beyotime, P2080S, China) according to the manufacturer’s protocol. Briefly,1*10^7^ cells were fixed with 1% paraformaldehyde for 10 min and scraped off. Then, cell pellets were collected after centrifugation and resuspended in 1 ml lysis buffer and sonicated. The lysates were centrifugation to remove cell debris, and the chromatin solutions were diluted. Chromatin fragments were immunoprecipitated with primary antibody or IgG overnight at 4 °C. Then, Protein A/G Magnetic Beads were added to the lysate and incubated at 4 °C for 2 h. The DNA fragments were purified with a DNA Purification Kit (Beyotime, D0033, China) after reverse cross-linking at 65 °C for 4 h. The immunoprecipitated DNA was analyzed by qPCR normalized to IgG control.

### Luciferase reporter assay

The reporter plasmids containing the HK2 promoter site2, as well as matched mutant sequences (CAAGTCCCTTC), were designed by GeneChem (Shanghai, China). Briefly, 293T cells were transfected with the indicated plasmids. 48 h after transfection, the firefly luciferase activity was calculated and analyzed.

### Statistics

Survival analysis was performed using univariate and multivariate Cox regression analyses, and survival curves were plotted with the Kaplan–Meier method. Comparisons between groups were conducted using Student’s t test or one-way ANOVA. All in vitro experiments were performed in triplicate and repeated three times. Statistical analysis was performed using GraphPad Prism 9.0.0 and SPSS (version 20.0). *p* < 0.05 was considered statistically significant, and the data are presented as means ± standard deviation. *P < 0.05; **P < 0.01; ***P < 0.001.

## Result

### CCT6A was highly expressed in LUAD and correlated with worse prognosis

**Fig. 1:**
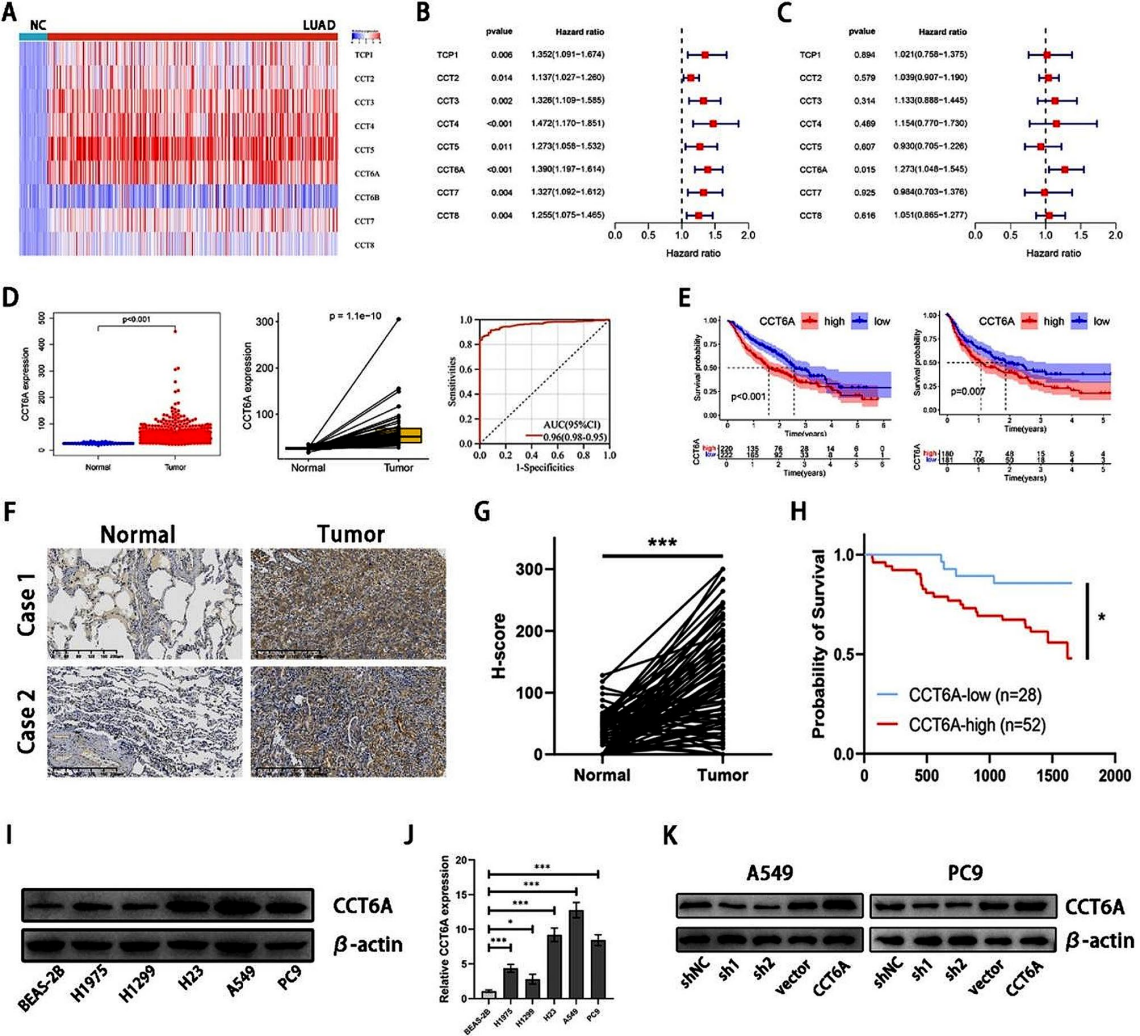
CCT6A was highly expressed in LUAD and negatively correlated with the prognosis. **(A)** Heatmap of the CCT family gene expression in LUAD based on TCGA. **(B)** Univariate Cox analysis of the prognosis-related CCT family genes based on TCGA. **(C)** Multivariate Cox analysis of the prognosis-related CCT family genes based on TCGA. **(D)** Unpaired (left) and paired (middle) t tests of CCT6A expression between LUAD and normal tissues and ROC curve showing the sensitivity and specificity to predict the occurrence of LUAD (right) based on TCGA. **(E)** Kaplan Meier plots showing the significant difference of OS (left) and PFS (right) in LUAD patients between the CCT6A-high and CCT6A-low samples based on GSE68465. **(F)** Representative IHC plots of tumor and paired normal tissues (20×magnification). Scale bars = 200 μm. **(G)** Paired t tests of the H-score between tumor and normal tissues. **(H)** Kaplan Meier plots showing the significant difference of OS in LUAD patients with CCT6A-high and CCT6A-low tissues. **(I)** CCT6A protein expression in normal lung epithelial cell line and different LUAD cell lines. **(J)** CCT6A mRNA expression in normal lung epithelial cell line and different LUAD cell lines. **(K)** The transfection efficiency of shRNAs and plasmid in A549 and PC9 cell lines

To explore the roles of CCT family genes in LUAD, we conducted differential expression and Cox proportional hazards analyses using TCGA database. Among the nine investigated genes, all except CCT6B showed higher expression levels in LUAD tissues than in normal tissues (Fig. 1A) and were correlated with poorer prognosis of LUAD patients (Fig. 1B). Further, multivariate Cox analysis indicated CCT6A as an independent prognostic factor for LUAD, linked to worse survival outcomes (Fig. 1C).

Based on TCGA-LUAD, the expression of CCT6A in LUAD tissues was significantly elevated compared to that in normal tissues, indicating its high specificity and sensitivity in distinguishing LUAD (Fig. 1D). Data from GSE68465 demonstrated that patients with lower CCT6A expression had improved overall survival (OS) and progression-free survival (PFS) than those with higher expression levels (Fig. 1E). CCT6A expression also increased in more advanced TNM stages (Figure S1). A total of 80 pairs of LUAD tumors and adjacent normal tissues were collected, with their clinical information summarized in Table 1. Immunohistochemistry (IHC) staining showed a significant increase in CCT6A protein levels in LUAD tissues versus normal tissues (Fig. 1F-G), and patients with higher H-scores experienced significantly worse prognoses (Fig. 1H). Western blot (WB) and PCR analyses confirmed higher CCT6A expression in LUAD cell lines compared to that in normal pulmonary epithelial cell line (Fig. 1I-J).

**Table 1:** The clinical information of the patients

Variants	Total [Cases (%)]	CCT6A Expression [Cases (%)]		*P* value
		Low	High	
**Total**	80	28	52	
**Gender**				
Male	36 (45%)	16 (57.14%)	30 (57.69)	0.999
Female	44 (55%)	12 (42.86%)	22 (42.14)	
**Age at diagnosis**				
>65	19 (23.75%)	4 (14.29%)	15 (28.85%)	0.177
≤ 65	61 (76.25%)	24 (85.71%)	37 (71.15%)	
**Pathology stage**				
I	19 (23.75%)	9 (32.14%)	8 (15.38%)	0.078
II	23 (28.75%)	11 (39.29%)	14 (26.92%)	
III	24 (30%)	6 (21.43%)	19 (36.54%)	
IV	13 (16.25%)	2 (7.14%)	11 (21.15%)	
**Tumor size**				
≤ 3 cm	20 (25%)	13 (46.43%)	12 (23.08)	0.044
>3 cm	60 (75%)	15 (53.57%)	40 (76.92)	
**Lymph node metastasis**				
Absent	31 (38.75%)	14 (50%)	18 (34.62%)	0.233
Present	49 (61.25%)	14 (50%)	34 (65.38%)	

### CCT6A knockdown impaired LUAD proliferation both in vitro and in vivo

**Fig. 2:**
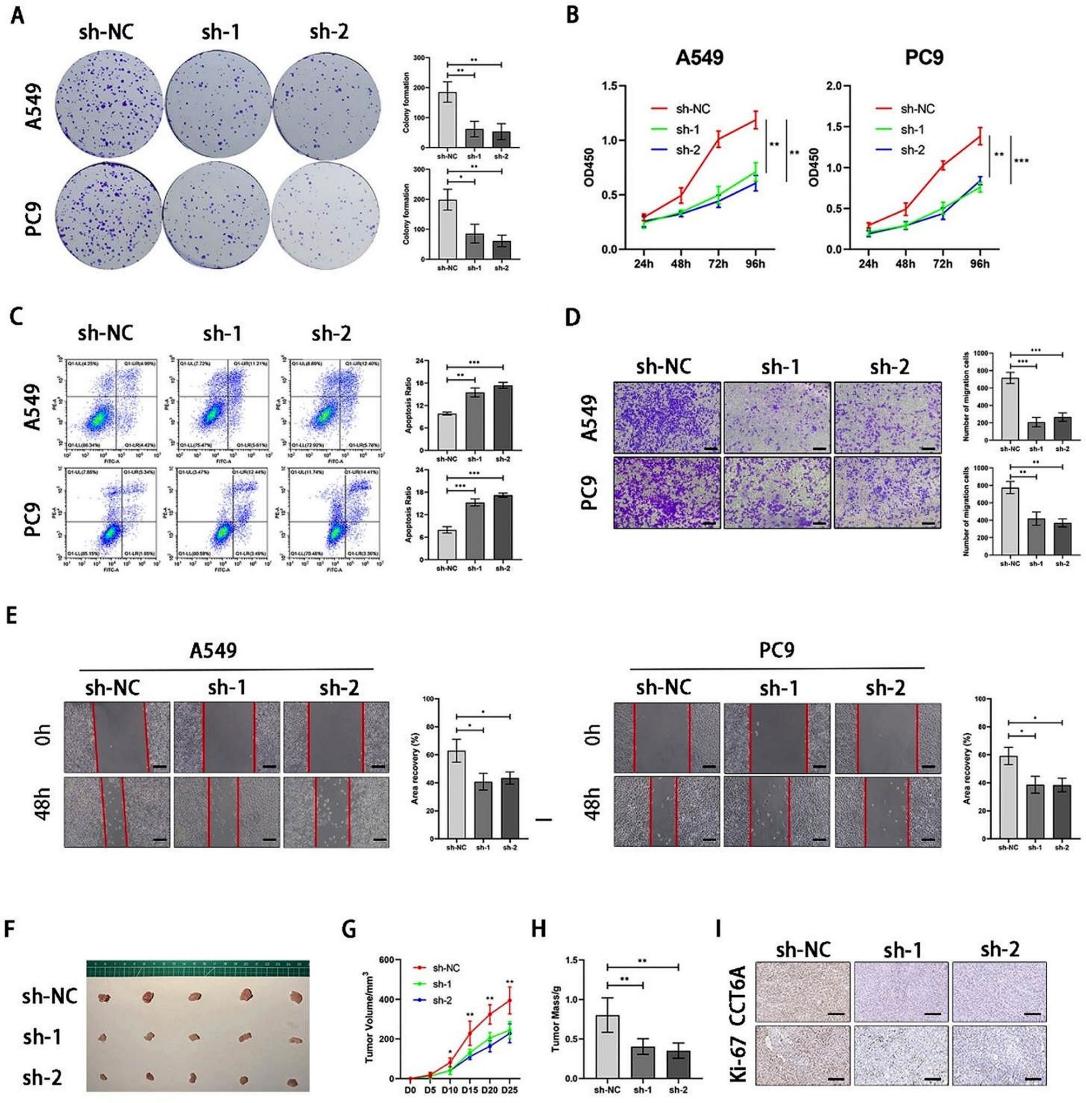
Loss of CCT6A inhibits the proliferation of LUAD in vitro and in vivo **(A-B)** Colony formation assays and growth curves (days 1–4) represented the proliferation of A549/PC9 cells infected with sh-NC or sh-CCT6A. Representative images of the crystal violet staining of cells in a 6-well plate and statistics were shown. **(C)** CCT6A knockdown resulted in increased apoptosis in LUAD cells. Representative FACS images and statistics based on three independent experiments were shown. **(D)** Transwell assays were performed to assess the migration of A549/PC9 cells infected with sh-NC or CCT6A-sh-1/2. Cells crossing the membrane were dyed with crystal violet (10×magnification). Scale bars = 100 μm. **(E)** Migration of A549/PC9 cells with CCT6A knockdown was assessed using wound healing assays. Area coverages were observed at 48 h (10× magnification). Scale bars = 100 μm. **(F)** Representative images of subcutaneous tumors on the left flank of mice injected with sh-NC or sh-CCT6A A549 cell (*n* = 5, each group). **(G)** Tumor growth curves were plotted every 5 days to 25days. **(H)** The weight of tumors was measured. **(I)** Representative images of IHC staining for CCT6A and Ki67 proteins (20×magnification). Scale bars = 100 μm. * *P* < 0.05; ** *P* < 0.01; *** *P* < 0.001. Variables are presented as mean ± SD

In the A549 and PC9 cell lines, CCT6A expression was either decreased through transfection with short hairpin RNAs (shRNAs) or increased via plasmid transfection (Fig. 1K). Notably, CCT6A silencing suppressed both colony formation and proliferation in LUAD cell lines (Fig. 2A-B). This intervention also resulted in a substantial increase in the proportion of apoptotic cells (Fig. 2C). Further analysis using scratch wound healing and transwell assays demonstrated that CCT6A silencing markedly reduced LUAD cell migration compared to control groups (Fig. 2D-E). The effect of CCT6A on LUAD’s tumorigenic potential was additionally assessed in vivo through the subcutaneous injection of A549 cells into BALB/c-nude mice (Fig. 2F), which showed that CCT6A knockdown significantly slowed tumor volume and weight growth (Fig. 2G-H). Moreover, CCT6A knockdown was observed to decrease the Ki-67 index, indicating a reduction in tumor cell proliferation (Fig. 2I).

Conversely, overexpressing CCT6A facilitates LUAD cell growth, proliferation, and migration while reducing the proportion of apoptotic cells compared to the control group (Figure S2A-E). This was accompanied by increased tumorigenicity in vivo and a higher Ki-67 index (Figure S2F-I). These findings collectively confirm CCT6A’s critical role in enhancing tumor growth and proliferation, both in vitro and in vivo.

### CCT6A enhanced aerobic glycolysis in LUAD cells

**Fig. 3:**
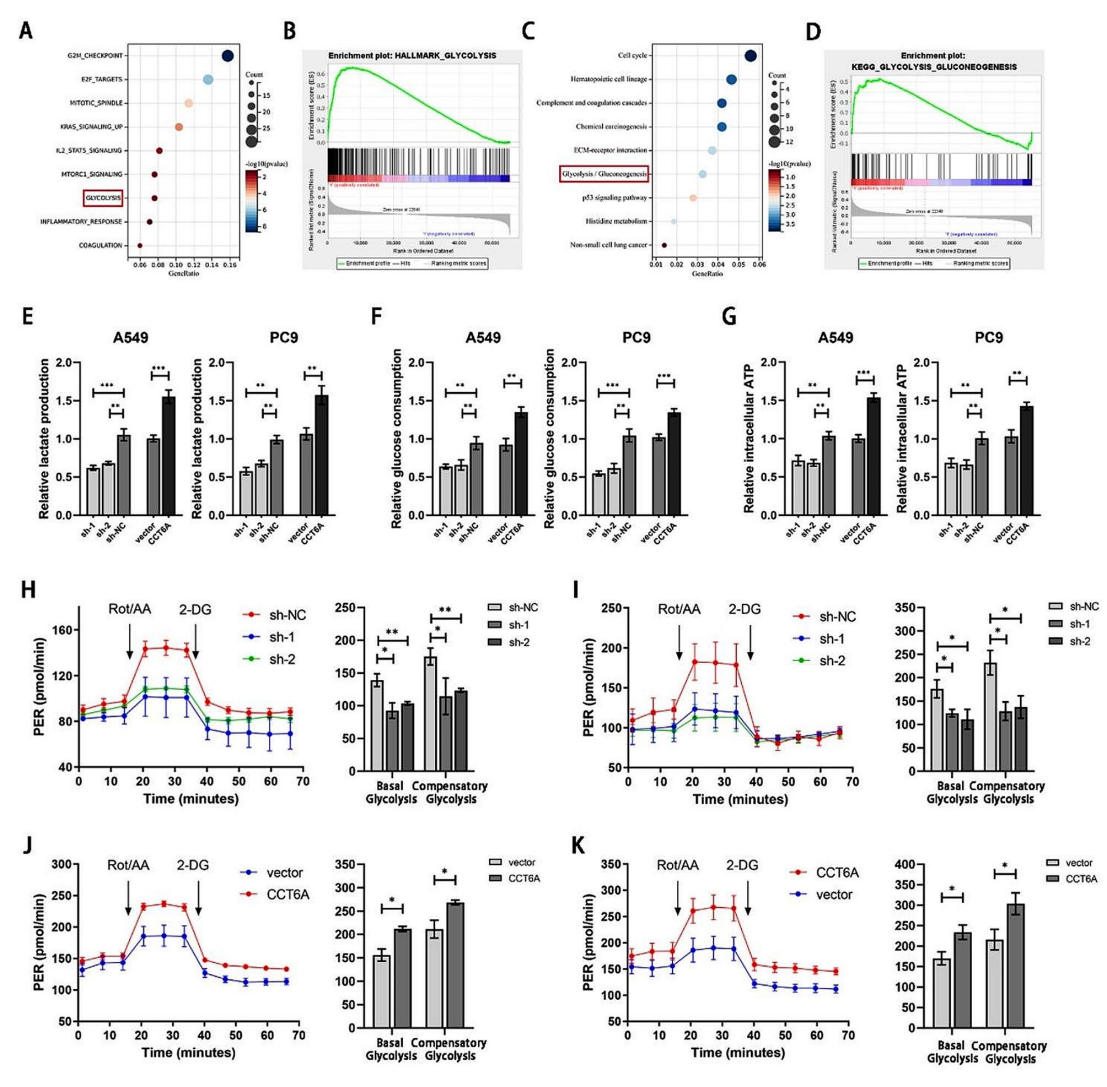
CCT6A promoted glycolysis of LUAD. **(A)** Bubble plot of enriched hallmark pathways based on differential genes between CCT6A-high and CCT6A-low samples. **(B)** GSEA plot of hallmark-glycolysis pathway based on CCT6A expression. **(C)** Bubble plot of enriched KEGG pathways based on differential genes between CCT6A-high and CCT6A-low samples. **(D)** GSEA plot of KEGG-glycolysis-gluconeogenesis pathway based on CCT6A expression. **(E-G)** The impact of CCT6A knockdown and overexpression on lactate production, glucose consumption, and intracellular ATP in A549/PC9 cells after 24 h was evaluated. **(H-K)** Proton efflux rate (PER) in CCT6A knockdown and overexpression A549/PC9 cells. Basal glycolysis and compensatory glycolysis were quantified and shown as histograms. * *P* < 0.05; ** *P* < 0.01; *** *P* < 0.001. Variables are presented as mean ± SD

To unravel the underlying mechanism by which CCT6A promotes LUAD cell proliferation and migration, we conducted pathway enrichment analysis using the TCGA database. Remarkably, the glycolysis pathway was enriched in the CCT6A-high group, as indicated by analyses using both HALLMARK (Fig. 3A-B) and KEGG (Fig. 3C-D) gene sets. Further exploration into CCT6A’s impact on glucose metabolism revealed that CCT6A knockdown led to decreases in lactate production, glucose consumption, and intracellular ATP level in A549 and PC9 cells, while CCT6A overexpression produced the opposite effects (Fig. 3E–G). Analysis of proton efflux rate (PER), which reflects extracellular acidification rate, provided additional evidence that CCT6A silencing diminished the glycolytic capacity of LUAD cells (Fig. 3H-I). Conversely, CCT6A overexpression enhanced this capacity (Fig. 3J-K).

### CCT6A orchestrated the oncogenic and glycolytic functions in LUAD through HK2

**Fig. 4:**
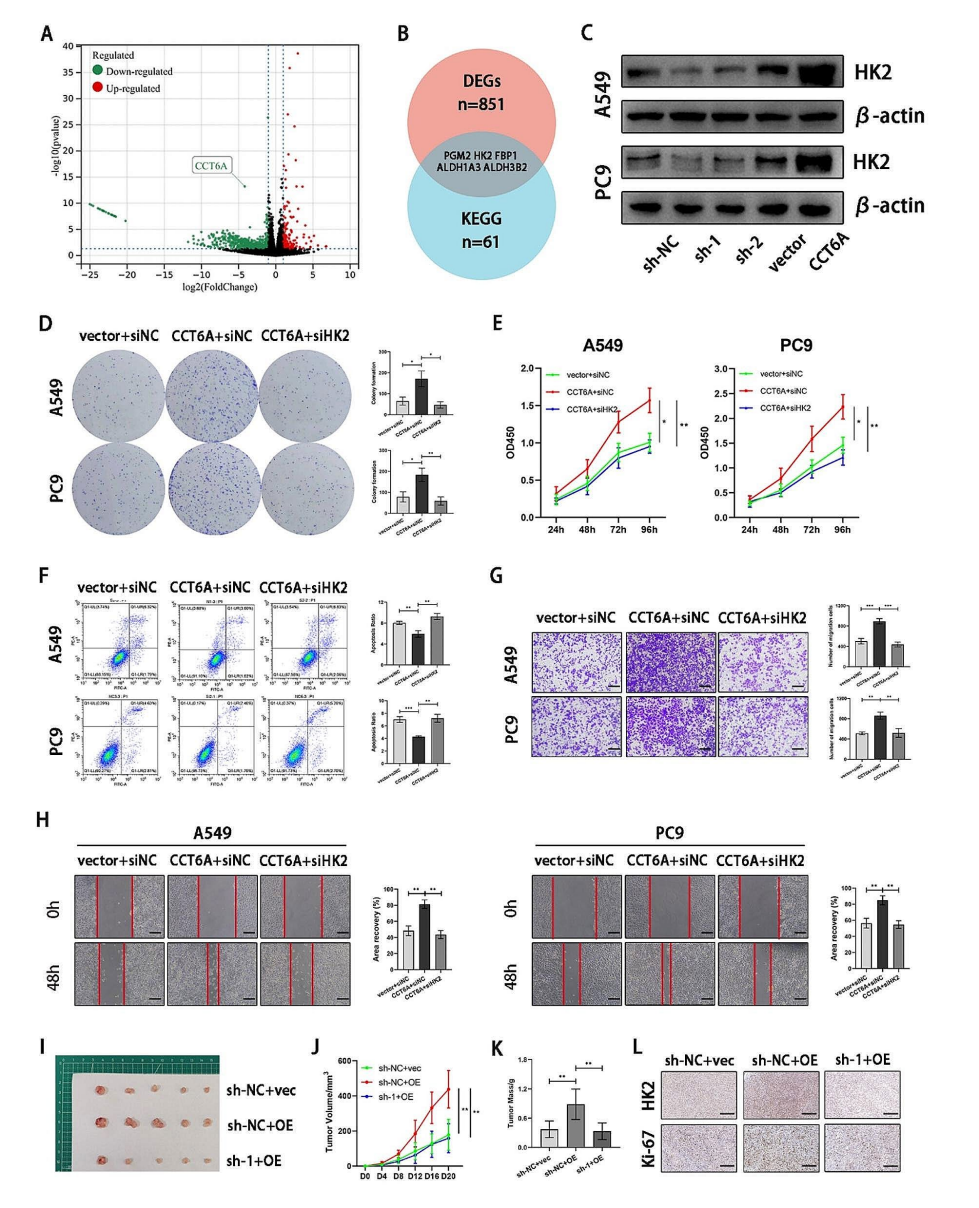
Oncogenic role of CCT6A depended on HK2. **(A)** Volcano plot of genes significantly downregulated or upregulated upon CCT6A knockdown. **(B)** Venn diagram of differential genes (|log_2_FC|>1, *P* < 0.05) upon CCT6A knockdown and associated with the glycolysis metabolism. **(C)** HK2 expression in cells infected with sh-NC, sh-CCT6A, vector, and CCT6A-OE plasmid were shown by WB. **(D-E)** Colony formation assays and growth curves (days 1–4) represented the proliferation of A549/PC9 cells treated with vector or CCT6A-OE plasmid plus siNC/siHK2, Representative images of the crystal violet staining of cells in a 6-well plate and statistics were shown. **(F)** HK2 knockdown reversed the deceased apoptosis induced by CCT6A overexpression. Representative FACS images and statistics based on three independent experiments were shown. **(G)** Transwell assays showed migration of vector/CCT6A-overexpression infected A549/PC9 cells with siNC/siHK2 (10×magnification). Scale bars = 100 μm. **(H)** Scratch wound healing represented the migration of A549/PC9 cells treated with vector or CCT6A-OE plasmid plus siNC/siHK2 (10× magnification). Scale bars = 100 μm. **(I)** Representative images of subcutaneous tumors on the left flank of mice injected with A549/PC9 cells treated with sh-NC or sh-1 plus vector/HK2-OE plasmid (*n* = 5, each group). **(J)** Tumor growth curves were plotted every 4 days to 20days. **(K)** The weight of tumors was measured. **(L)** Representative images of IHC staining for HK2 and Ki67 proteins (20×magnification). Scale bars = 100 μm. * *P* < 0.05; ** *P* < 0.01; *** *P* < 0.001. Variables are presented as mean ± SD

To investigate the mechanism through which CCT6A facilitates the progression and glycolysis in LUAD cell lines, we performed RNA sequencing (RNA-seq) analysis on A549 cells with CCT6A knockdown. Totally, 851 differentially expressed genes (DEGs) were identified (Fig. 4A), including five associated with glycolysis (Fig. 4B). Among these, HK2 was notable for its direct involvement in the glycolytic pathway as a rate-limiting enzyme. HK2 was found to be highly expressed in LUAD tissues compared to normal tissues, and elevated HK2 expression correlated with poorer OS of LUAD patients (Figure S3A-B). CCT6A knockdown led to reduced HK2 protein expression, whereas CCT6A overexpression resulted in increased HK2 levels (Fig. 4C). Besides, the enhancement of colony formation and proliferation in LUAD cell lines induced by CCT6A overexpression were attenuated by HK2 knockdown (Fig. 4D-E). HK2 knockdown also reversed the reduction in apoptosis observed with CCT6A overexpression (Fig. 4F). Scratch wound healing and transwell assay demonstrated that HK2 knockdown prevented the LUAD cell migration enhancement induced by CCT6A overexpression (Fig. 4G-H). Moreover, HK2 overexpression restored the compromised in vivo tumorigenesis capacity associated with CCT6A silencing (Fig. 4I-L).

**Fig. 5:**
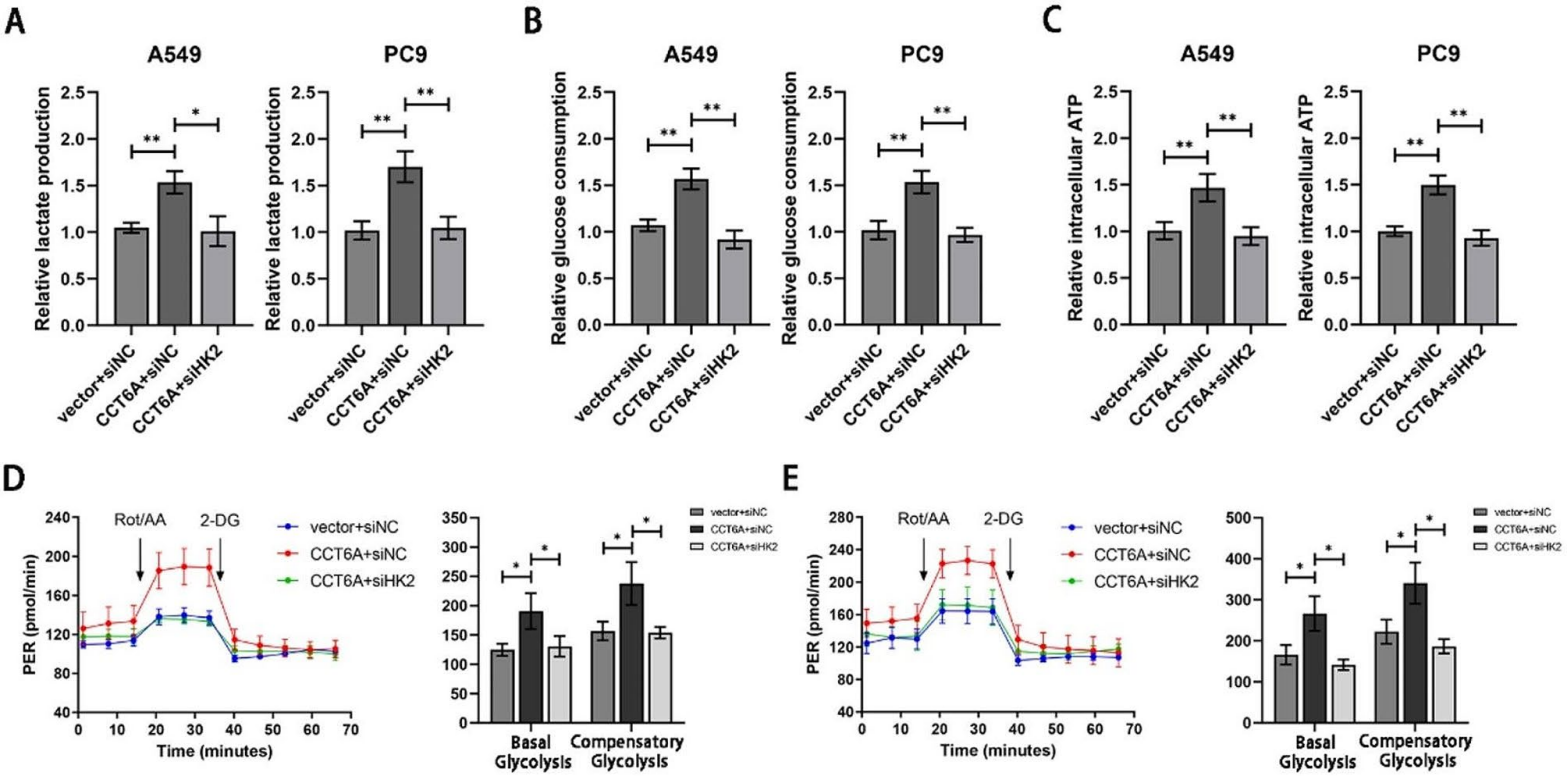
CCT6A promoted glycolysis in a HK2-dependent way. **(A-C)** Lactate production, glucose consumption, and intracellular ATP in A549/PC9 cells treated with vector or CCT6A-OE plasmid plus siNC/siHK2 was evaluated. **(D-E)** Proton efflux rate (PER) in A549/PC9 cells treated with vector or CCT6A-OE plasmid plus siNC/siHK2. Basal glycolysis and compensatory glycolysis were quantified and shown as histograms. * *P* < 0.05; ** *P* < 0.01; *** *P* < 0.001. Variables are presented as mean ± SD

Following HK2 knockdown, lactate production, glucose consumption, and intracellular ATP level significantly decreased in A549 and PC9 cells that had undergone CCT6A overexpression (Fig. 5A-C). Additionally, the glycolytic capacity in these cells was diminished with HK2 silencing (Fig. 5D-E).

### CCT6A enhanced the protein stability of STAT1 via suppressing ubiquitination

**Fig. 6:**
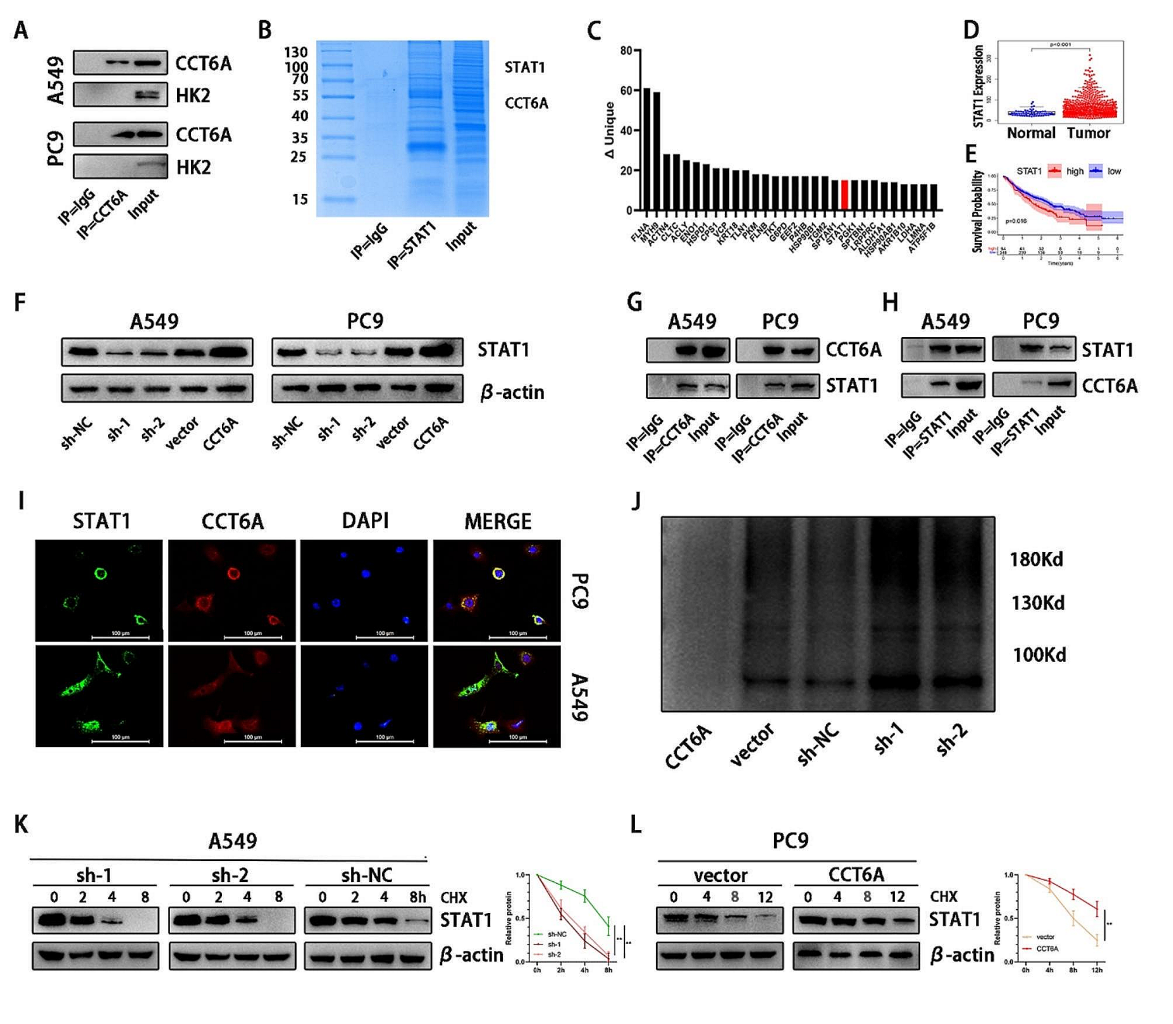
CCT6A bound and stabilized STAT1 protein. **(A)** Immunoprecipitation of cell lysates from A549/PC9 cells using anti-CCT6A or IgG antibodies, and immunoprecipitants were blotted with anti-CCT6A or anti-HK2 antibodies. **(B)** Coomassie blue staining of proteins immunoprecipitated by anti-CCT6A or IgG antibodies and input. **(C)** Bar plot showing proteins specifically binding with CCT6A. **(D)** Unpaired t tests of STAT1 expression between LUAD and normal tissues based on TCGA. **(E)** Kaplan Meier plots showing the significant difference of OS in LUAD patients between the STAT1-high and STAT1-low samples based on TCGA. **(F)** STAT1 expression in A549/PC9 cells infected with sh-NC/sh-CCT6A or vector/CCT6A-OE plasmid shown by WB. **(G-H)** Immunoprecipitation of cell lysates from A549/PC9 cells using anti-CCT6A, anti-STAT1 or IgG antibodies, and immunoprecipitants were blotted with anti-STAT1 or anti-CCT6A antibodies. **(I)** Immunofluorescence images showing the distribution of CCT6A (red) and STAT1 (green) in A549/PC9 cells (40×magnification). Scale bars = 100 μm. **(J)** After 48 h transfection, A549 cells overexpressed CCT6A were treated with MG132 for 8 h and followed by IP and western blot. **(K)** A549 cells infected with CCT6A knockdown were treated with CHX for indicated time and immunoblotting analysis was performed. **(L)** PC9 cells infected with CCT6A overexpression were treated with CHX for indicated time and immunoblotting analysis was performed. * *P* < 0.05; ** *P* < 0.01; *** *P* < 0.001. Variables are presented as mean ± SD

Given established role of the CCT family in protein binding and stabilization, we aimed to explore whether CCT6A could enhance HK2 expression through this mechanism. Interestingly, CCT6A was not found to directly interact with HK2 (Fig. 6A), prompting us to consider that CCT6A might instead assist in the stabilization of a transcription factor, thereby facilitating its activity and leading to an increase in HK2 expression. To investigate this, mass spectrometry (MS) analysis was performed to identify transcription factors interacting with CCT6A (Fig. 6B). STAT1 emerged as a significant candidate, exhibiting higher expression levels in LUAD cells and correlating with shorter survival in LUAD patients (Fig. 6C-E). The protein levels of STAT1 were observed to decrease following CCT6A knockdown and increase upon CCT6A overexpression (Fig. 6F). Through co-immunoprecipitation (co-IP), we uncovered a binding interaction between CCT6A and STAT1 (Fig. 6G-H), which was further corroborated by confocal fluorescence microscopy demonstrating their co-localization in the cytoplasm (Fig. 6I). Notably, STAT1 expression did not exhibit a linear correlation with CCT6A expression (Figure S3C) and did not significantly change with CCT6A modulation (Figure S3D), indicating that CCT6A influences STAT1 post-transcriptionally.

Ubiquitination is a prevalent pathway for protein degradation, prompting us to investigate CCT6A’s impact on the ubiquitination of STAT1 protein. Silencing CCT6A was observed to increase the ubiquitin-bound fraction of STAT1, suggesting enhanced ubiquitin-mediated degradation. Conversely, overexpression of CCT6A produced opposing results (Fig. 6J). Additionally, we assessed the protein levels of STAT1 in LUAD cells with cycloheximide (CHX), a protein synthesis inhibitor. Consistent with expectations, CCT6A knockdown hastened the degradation of STAT1 (Fig. 6K) while overexpression of CCT6A decelerated this process (Fig. 6L). These findings suggested that CCT6A might augment the stability of STAT1 by diminishing its ubiquitination.

We also explored whether CCT6A’s regulation of STAT1 involves ubiquitin E3 ligases. TRIM21 was confirmed to act as a ubiquitin E3 ligase to ubiquitinate STAT1. TRIM21 still downregulated STAT1 regardless of whether CCT6A was knocked down or overexpressed (Figure S3E), indicating that CCT6A’s regulatory effect on STAT1 is not mediated through TRIM21. Furthermore, CCT6A did not alter the expression of TRIM21 (Figure S3F) nor was it able to recruit TRIM21 to STAT1 (Figure S3G). These results suggested that CCT6A modulates STAT1 stability in a manner that is independent of ubiquitin E3 ligase activity.

### STAT1 facilitated the transcription of HK2

**Fig. 7:**
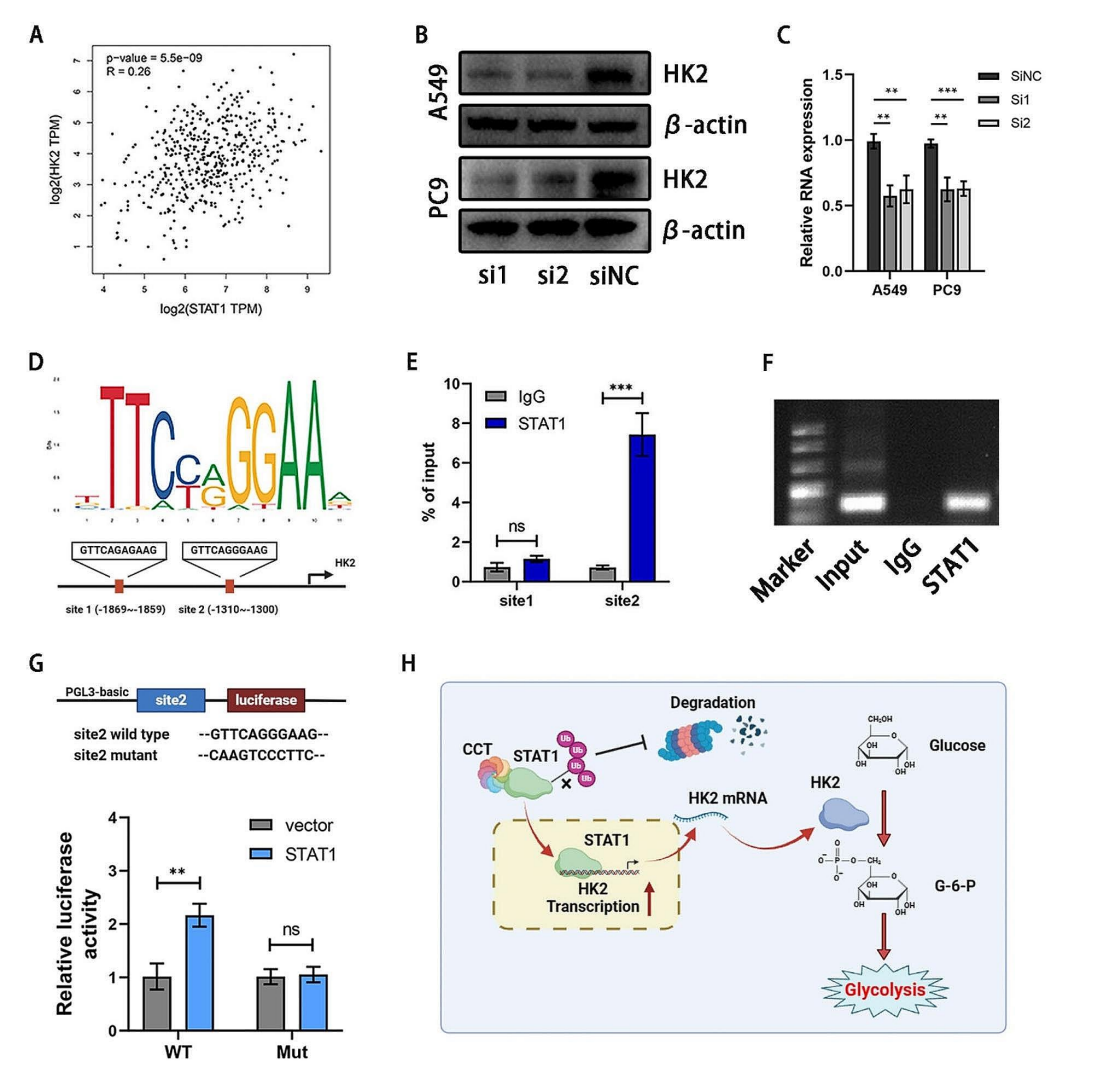
STAT1 facilitates the transcription of HK2. **(A)** STAT1 was positively correlated with the HK2 expression in LUAD cohort. **(B-C)** Western blot and PCR results suggested the HK2 expression decreased as STAT1 knockdown. **(D)** Bioinformation analysis of the promoter binding sites of STAT1. **(E)** ChIP assay results shown by qPCR indicated the binding of STAT1 on the site2 of HK2 promoter. **(F)** Agarose gel electrophoresis suggested STAT1 could bind HK2 promoter on site2. **(G)** Mut and WT sequences of the HK2 promoter site2 were respectively constructed. A luciferase reporter assay was performed to detect the luciferase activity. **(H)**) Schematic diagram of CCT6A/STAT1/ HK2 axis promoting the glycolysis in LUAD cells. * *P* < 0.05; ** *P* < 0.01; *** *P* < 0.001. Variables are presented as mean ± SD

Then, we sought to ascertain whether STAT1 could promote the transcription of HK2. Initially, we observed a correlation between the two mRNA levels (Fig. 7A). Upon silencing STAT1, both HK2 protein and mRNA levels decreased in LUAD cell lines (Fig. 7B-C). Employing online bioinformatics predictive tools (JASPAR, http://jaspar.genereg.net/), we identified two potential STAT1 binding sites within the promoter region of the HK2 gene (Fig. 7D). The binding relationship between STAT1 and the HK2 promoter was subsequently validated through a ChIP assay, affirming site2 as the functional binding site (Fig. 7E-F). To assess the specificity of this interaction, we mutated the HK2 promoter at site2, which significantly reduced STAT1-dependent transcriptional activation (Fig. 7G).

Collectively, CCT6A interacts with and stabilizes STAT1, inhibiting its ubiquitination degradation, and subsequently indirectly upregulates HK2 expression through STAT1’s transcriptional activity. This chain of events augments glycolysis, proliferation, and migration in LUAD cells.

## Discussion

The eukaryotic group II chaperonin CCT has been established to play important roles in protein folding [32, 33]. In the context of cancers, CCT is implicated in pro-oncogenic activities through their assistance in the folding of numerous oncogenes [34]. In this study, we aimed to elucidate the impact of CCT proteins on the progression of LUAD. Among the DEGs, CCT6A was identified as the only independent prognostic marker. It exhibited high expression levels in both LUAD cell lines and clinical tissues, with elevated expression correlating with a poorer Overall Survival (OS) and Progression-Free Survival (PFS) in LUAD patients. Silencing of CCT6A not only hindered the proliferation of LUAD cells but also induced apoptosis. Additionally, we validated the tumorigenic role of CCT6A in in vivo models. Pathway analysis and subsequent research into metabolism revealed that CCT6A enhances aerobic glycolysis, which in turn supports LUAD cell proliferation and migration.

Aerobic glycolysis, characterized by enhanced glucose uptake, and ATP and lactate production in conditions of sufficient oxygen, is critical for cancer cell proliferation, migration and resistance to treatment. Aerobic glycolysis accounts for up to 50–70% of total ATP supply in various tumors, despite its relative lower efficiency in ATP production [35]. The metabolic intermediates produced during this process are also crucial for the biosynthesis of biomacromolecules, which support rapid tumor growth. Lactate production also creates an acidic environment that might impair immune surveillance [36]. Additionally, heightened aerobic glycolysis might attribute to chemotherapy and tyrosine kinase inhibitor (TKI) resistance in lung cancer [37–39]. Considering these insights, presents a promising avenue for the development of new therapeutic strategies.

Subsequently, we focused on the downstream molecules of CCT6A that facilitate aerobic glycolysis. Through transcriptome sequencing analysis, we observed a marked decrease in the expression of hexokinase 2 (HK2), a pivotal rate-limiting enzyme, following the silencing of CCT6A. Hexokinases serve to catalyze the transformation of glucose into glucose-6-phosphate, thus initiating aerobic glycolysis. Among its isoforms—HK1, HK2, HK3, and HK4—HK2 stands out as the most effective in promoting aerobic glycolysis [40]. Previous research has illuminated its association with voltage-dependent anion-selective channel protein 1 (VDAC1) located in the mitochondrial outer membrane. This interaction leads to increased ATP production and reduced apoptosis by activating enzymes related to ATP synthesis [41]. Notably, HK2 expression is significantly higher in cancer cells than in normal cells, correlating with accelerated proliferation and enhanced resistance to drugs through metabolic reprogramming across various cancer types [28–31]. The expression of HK2 is regulated by various signaling pathways, including β-catenin/c-Myc and PI3K/Akt axes [42, 43], as well as transcription factors like HIF-1 and STAT3 [28, 44]. Our investigation additionally reveals that STAT1 plays a role in the transcriptional regulation of HK2, further delineating the complex network of molecular interactions driving cancer metabolism.

The Signal Transducer and Activator of Transcription (STAT) family consists of seven members: STAT1, STAT2, STAT3, STAT4, STAT5A, STAT5B, and STAT6. STAT1 has traditionally been regarded as a tumor-suppressor, inhibiting angiogenesis and metastasis, while promoting apoptosis and enhancing immunosurveillance [45–50]. However, recently study has unearthed an oncogenic potential for STAT1 in certain cancers [51, 52]. In our investigation, we demonstrate that CCT6A could bind to and stabilize the STAT1 protein, reducing its ubiquitination, and then promoted LUAD cell glycolysis, proliferation, and migration through the transcriptional upregulation of HK2.

Despite these findings, the study has certain limitations. Given that CCT complex helps folding nascent proteins to their functional conformation, representing the binding between CCT6A and nascent STAT1 protein was challenging, and more advanced technique was required to solve this problem. Additionally, the in vivo xenograft experiment had a relatively small sample size, and the glycolysis levels in animal tumors were not measured.

## Conclusion

In summary, our research established that CCT6A facilitated glycolysis, proliferation, and migration in LUAD. It enhanced the stability of the STAT1 protein, which in turn promoted the transcription of HK2. Consequently, the CCT6A/STAT1/HK2 axis emerged as a promising therapeutic target for LUAD.

### Electronic supplementary material

Below is the link to the electronic supplementary material.

Supplementary Material 1

## Data Availability

All data generated or analyzed during this study are included in this published article and its supplementary information files.

## Abbreviations

CCT6A: Chaperonin containing TCP1 subunit 6 A
LUAD: Lung adenocarcinoma
HK2: Hexokinase 2
TMA: Tissue microarray
PER: Proton efflux rate
CHIP: Chromatin immunoprecipitation
STAT: Signal transducer and activator of transcription
IHC: Immunohistochemistry
TKI: Tyrosine kinase inhibitor
