# Supplementary material for: CCT6A facilitates lung adenocarcinoma progression and glycolysis via STAT1/HK2 axis
Source: J Transl Med. 2024 May 15;22:460. doi: 10.1186/s12967-024-05284-7 (PMC11094951; doi:10.1186/s12967-024-05284-7)
Supplement: Supplementary file 1 — Supplementary Material 1 [file 12967_2024_5284_MOESM1_ESM.docx]

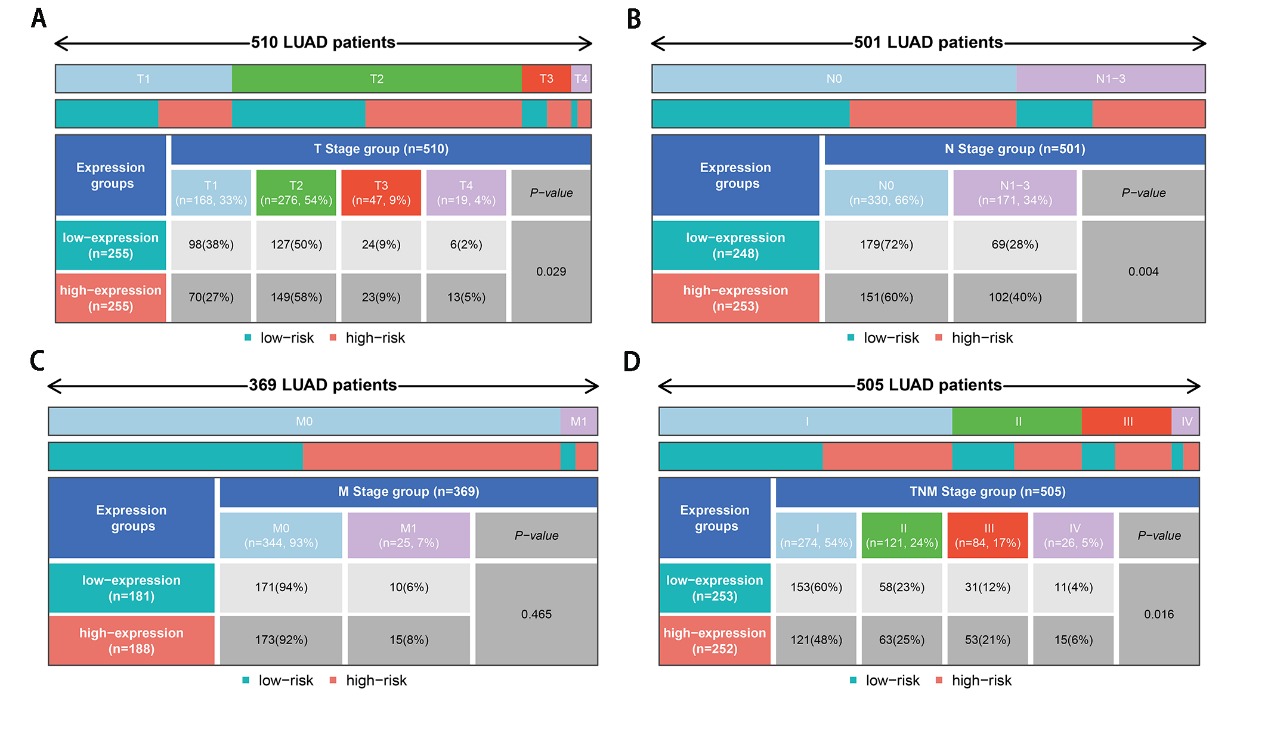


**Figure S1**: The correlation between CCT6A expression and T stage **(A)**, N stage **(B)**, M stage **(C)**, and TNM stage **(D)**.


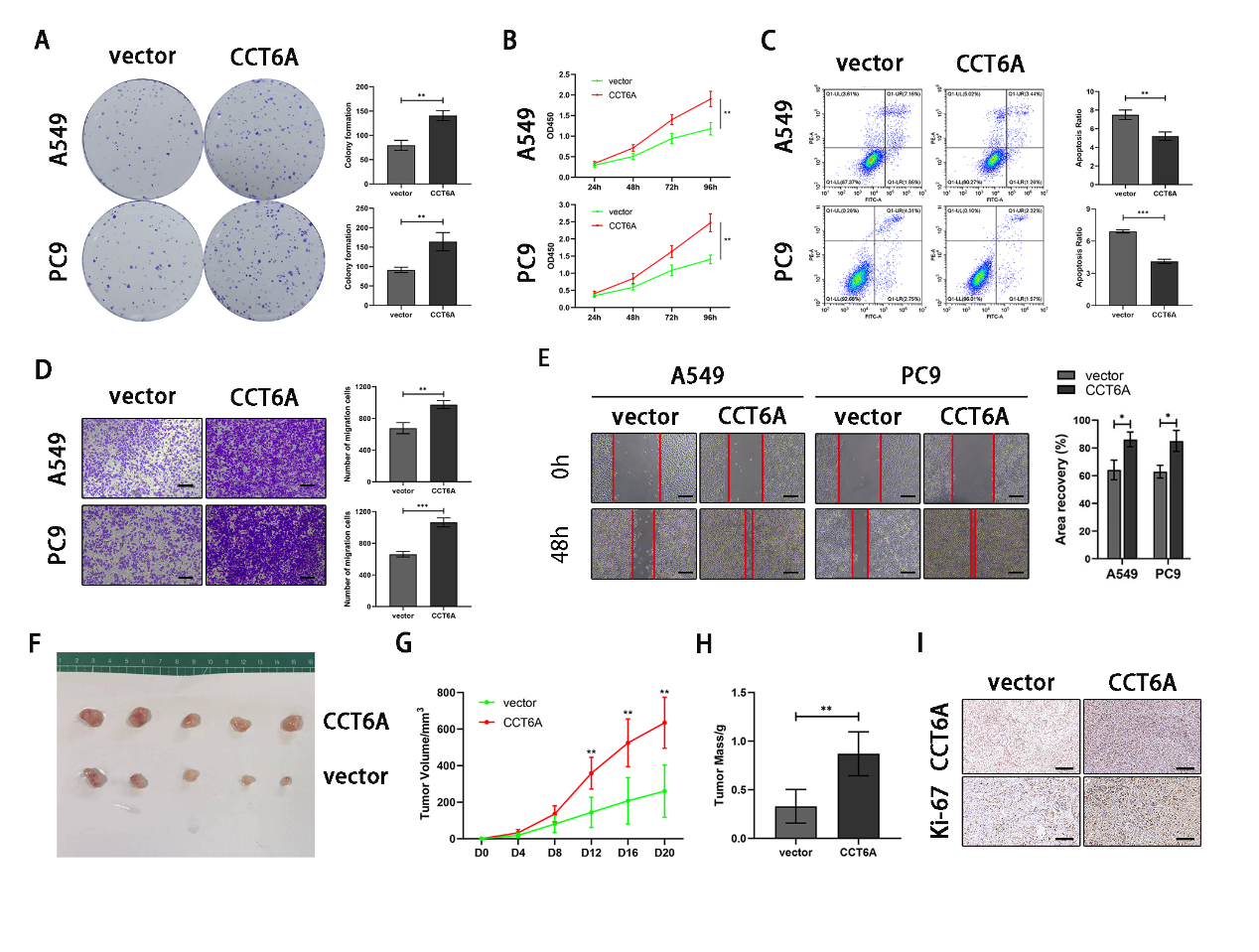


**Figure S2**: CCT6A promoted the proliferation of LUAD in vitro and in vivo.

**(A-B)** Colony formation assays and growth curves (days 1–4) represented the proliferation of A549/PC9 cells infected with vector or CCT6A-OE plasmid. Representative images of the crystal violet staining of cells in a 6-well plate and statistics were shown. **(C)** CCT6A overexpression resulted in decreased apoptosis in LUAD cells. Representative FACS images and statistics based on three independent experiments were shown. **(D-E)** Transwell and scratch wound healing assay represented the migration of A549/PC9 cells infected with vector or CCT6A-OE plasmid (10×magnification). Representative images of the crystal violet staining of cells in cell compartments and statistics were shown. Scale bars=100μm. **(F)** Representative images of subcutaneous tumors on the left flank of mice injected with vector or CCT6A overexpressed A549 cell (n = 5, each group). **(G)** Representative images of IHC staining for CCT6A and Ki67 proteins. (20×magnification). Scale bars=100μm. **(H)** Tumor growth curves were plotted every 6 days. **(I)** The weight of tumors was measured. * P < 0.05; ** P < 0.01; *** P < 0.001. Variables are presented as mean ± SD.


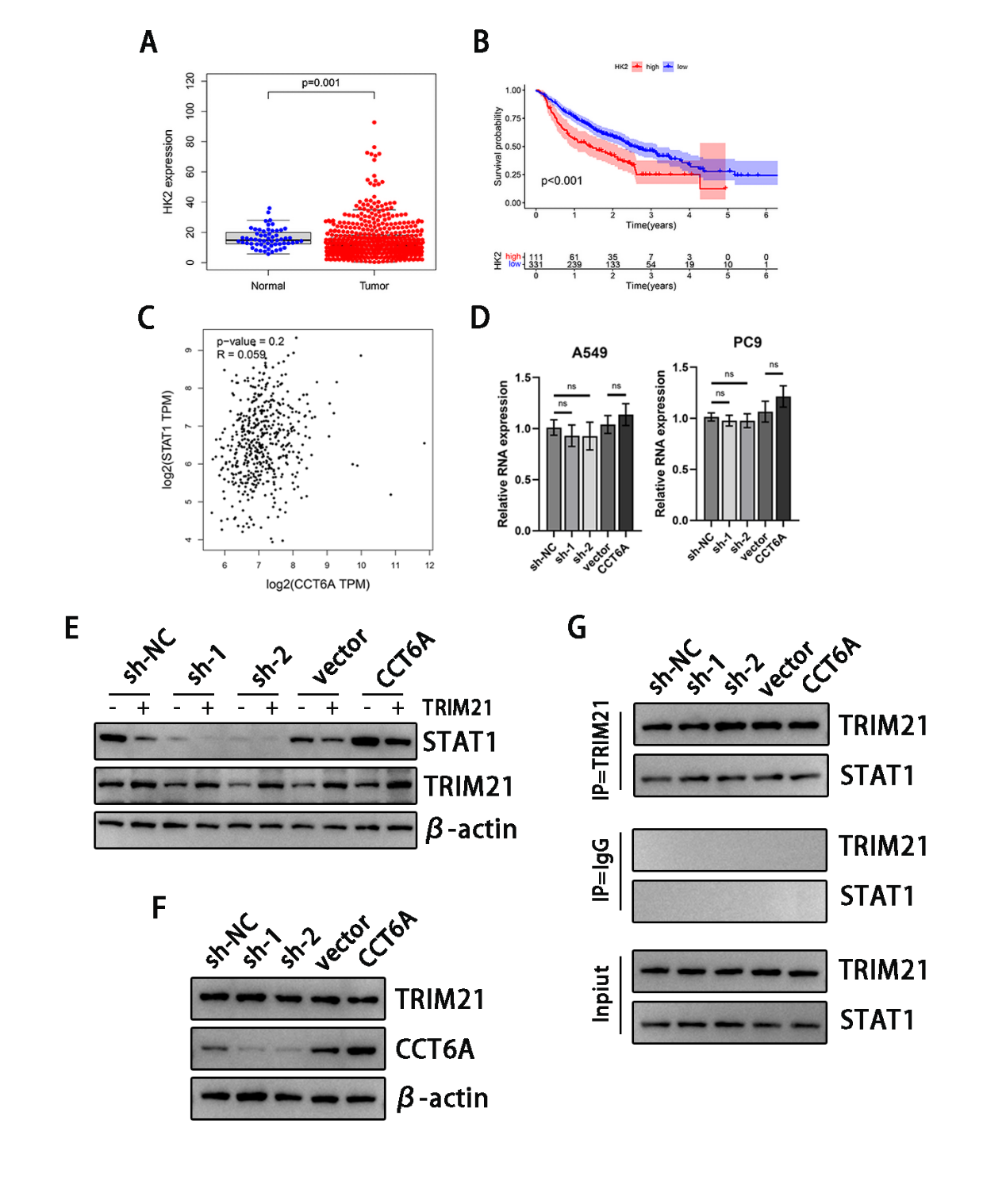


**Figure S3**: **(A)** Unpaired t tests of STAT1 expression between LUAD and normal tissues based on TCGA. **(B)** Kaplan Meier plots showing the significant difference of OS in LUAD patients between the STAT1-high and STAT1-low samples based on TCGA. **(C)** CCT6A showed none correlation with the HK2 expression in LUAD cohort. **(D)** PCR results suggested no notable change of STAT1 expression as CCT6A knockdown. **(E)** TRIM21 was not involved in the CCT6A-mediated regulation of STAT1, as measured in A549 cells with or without TRIM21 overexpression. **(F)** CCT6A did not affect TRIM21 expression, as measured by WB in A549 cells. **(G)** CCT6A did not influence the interaction between TRIM21 and STAT1, as measured by co-IP in A549 cells. * P < 0.05; ** P < 0.01; *** P < 0.001. Variables are presented as mean ± SD.

**Table S1**: The sequence used in the study.
